# What work has to be done to implement collaborative care for depression? Process evaluation of a trial utilizing the Normalization Process Model

**DOI:** 10.1186/1748-5908-5-15

**Published:** 2010-02-10

**Authors:** Linda Gask, Peter Bower, Karina Lovell, Diane Escott, Janine Archer, Simon Gilbody, Annette J Lankshear, Angela E Simpson, David A Richards

**Affiliations:** 1 National Primary Care Research and Development Centre, University of Manchester, Oxford Road, Manchester UK; 2 School of Nursing, Midwifery and Social Work, University of Manchester, Oxford Road, Manchester, UK; 3 Department of Health Sciences, Hull York Medical School (HYMS), Seebohm Rowntree Building, University of York, York, UK; 4 Cardiff School of Nursing and Midwifery Studies, Cardiff University, Caerleon Campus, Cardiff, UK; 5 School of Psychology, University of Exeter, Washington Singer Building, Perry Road, Exeter, UK

## Abstract

**Background:**

There is a considerable evidence base for 'collaborative care' as a method to improve quality of care for depression, but an acknowledged gap between efficacy and implementation. This study utilises the Normalisation Process Model (NPM) to inform the process of implementation of collaborative care in both a future full-scale trial, and the wider health economy.

**Methods:**

Application of the NPM to qualitative data collected in both focus groups and one-to-one interviews before and after an exploratory randomised controlled trial of a collaborative model of care for depression.

**Results:**

Findings are presented as they relate to the four factors of the NPM (interactional workability, relational integration, skill-set workability, and contextual integration) and a number of necessary tasks are identified. Using the model, it was possible to observe that predictions about necessary work to implement collaborative care that could be made from analysis of the pre-trial data relating to the four different factors of the NPM were indeed borne out in the post-trial data. However, additional insights were gained from the post-trial interview participants who, unlike those interviewed before the trial, had direct experience of a novel intervention. The professional freedom enjoyed by more senior mental health workers may work both for and against normalisation of collaborative care as those who wish to adopt new ways of working have the freedom to change their practice but are not obliged to do so.

**Conclusions:**

The NPM provides a useful structure for both guiding and analysing the process by which an intervention is optimized for testing in a larger scale trial or for subsequent full-scale implementation.

## Background

There is now a considerable evidence base for collaborative care as a 'technology' in the broadest sense for improving quality of care depression in the community [1,2], but an acknowledged gap between demonstrated efficacy of this novel intervention in randomised controlled trials and implementation in everyday practice [3]. Gunn and her colleagues [4] have described collaborative care for depression as a 'system level' intervention with four key characteristics:

1. A multi-professional approach to patient care: This requires that a general practitioner (GP) or family physician and at least one other health professional (*e.g*., nurse, psychologist, psychiatrist, pharmacist) are involved with patient care.

2. A structured management plan: in the form of guidelines or protocols: Interventions may include both pharmacological (*e.g*., antidepressant medication) and non-pharmacological interventions (*e.g*., patient screening, patient and provider education, counselling, cognitive behaviour therapy).

3. Scheduled patient follow-up: An organised approach to patient follow-up by systematically contacting patients to provide specific interventions, facilitate treatment adherence, or monitor symptoms or adverse effects.

4. Enhanced inter-professional communication: By introducing mechanisms to facilitate communication between professionals caring for the depressed person. This might include team meetings, case conferences, individual consultation/supervision, shared medical records, patient-specific written or verbal feedback between caregivers.

In the United Kingdom (UK) the Medical Research Council (MRC) guideline for the evaluation of complex interventions provided a phased methodological framework [5] highlighting the need for evaluation of process, which is essential for understanding the problems of integration of interventions into healthcare settings. Application of the framework suggested exploration of barriers and facilitators to implementation, which is an approach that has now been extensively used to understand the difficulties in implementation of collaborative care in the United States (US) [6-9]. However, the methodology used in these analyses has also been largely pragmatic, with only limited use of theoretical models to either interpret their findings or develop hypotheses for future research. The revised MRC Framework published in 2007 [10] emphasized the iterative nature of the tasks of defining and understanding the problem and its context, developing and optimizing and then evaluating the intervention, rather than viewing these as distinct conceptual stages. The utility of theoretical models drawing on health psychology (if the problem to be tackled is individuals' health behaviour) or social and organisational theory (to understand health service and practitioner factors) was specifically highlighted in this iteration of the framework, however the process by which an intervention is optimized for testing in a larger scale trial or for subsequent wide scale implementation remains *ad hoc*, with no clear framework to guide the researcher or future service developer.

Recently, May [11] proposed that the Normalization Process Model (NPM--see Table 1) provides a theoretical framework for understanding the workability (capable of being put into operation) and integration (assimilation into practice) of a complex intervention and demonstrated how this can used to understand trial outcomes [12]. Normalisation is concerned with the routine embedding of a classification, artefact, technique, or organisation practice in everyday work, and the NPM is specifically concerned with the work that people do to make a complex intervention work in everyday practice. It is therefore complementary to diffusion theory [13,14], which is concerned with the diffusion of innovation across networks, and psychological theories [15,16] that are concerned with intention and individual behaviour that might dispose professionals to adopt an intervention. May and colleagues have suggested that the NPM might be used to assess the normalization potential of a working practice (see table 1).

**Table 1:** Normalization Process Model from May *et al*. 2007

The collective action and interactions of patients, professionals and others are governed by four factors. We have derived questions from these factors as follows:	
**(i) Interactional workability**: This refers to how work is enacted by the people doing it. A complex intervention will affect co-operative interaction over work (congruence), and the normal pattern of outcomes of this work (disposal).	How does collaborative care for depression (CCD) impact on basic communication, clinical care and treatment at the level of patient and professional?
**(ii) Relational integration**: This refers to how work is understood within the networks of people around it. A complex intervention will affect not only the knowledge required by its users (accountability), but also the ways that they understand the actions of people around them (confidence).	How does CCD impact on the way that health professionals relate to each other? Does it seem to be the right thing to be doing? It is perceived as valid and/or useful? Who needs to be involved in the work? How do we inform them and link with them?
**(iii) Skill-set workability**: This refers to the place of work in a division of labor. A complex intervention will affect the ways that work is defined and distributed (allocation), and the ways in which it is undertaken and evaluated (performance).	Does this mean health professionals learning new skills or doing things differently? Is there a person available with the right set of skills to implement CCD? Does CCD challenge professional autonomy over working practices? Does it impact on case load and allocation of work?
**(iv) Contextual integration**: This refers to the organizational sponsorship and control of work. A complex intervention will affect the mechanisms that link work to existing structures and procedures (execution), and for allocating and organizing resources for them (realization)	Who has the power to make CCD happen? Does the system want it to happen? How can we divert resources to it?

### Brief description of the collaborative care trial

**Figure 1:**
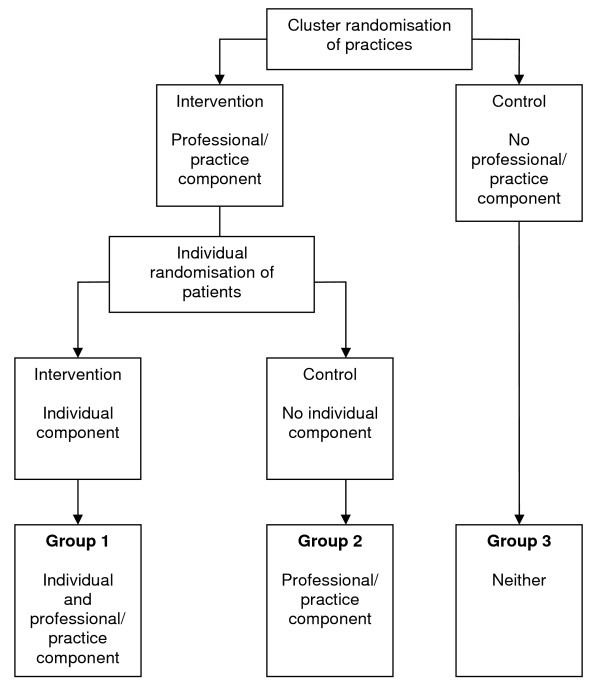
**design of main trial**.

As part of an exploratory trial of collaborative care for depression in a UK setting [17-19], we carried out a process evaluation to explore how the intervention might be adapted and made to work optimally in practice. The study team undertook a Phase II patient-level randomized controlled trial in primary care [18], nested within a cluster-randomized trial (this was in order to determine whether cluster- or patient-randomization would be the most appropriate design for a Phase III clinical trial--see Figure 1). The trial used an innovative design to determine the existence of contamination, as well as the effect of the collaborative care intervention.

Collaborative care includes a component that impacts on the individual patient (e.g., medication management from the case manager) and a component that impacts on the professionals and the practice (e.g., feedback of patient information to the GP, changes in practice organisation). In a standard, individually randomised trial, the component that impacts on the professional and the practice can lead to contamination, because it may influence patients in the control group. This trial used an individually randomised trial nested within a cluster trial. The design enables an analysis of the effect of the whole collaborative care intervention (through a comparison of group one and group three), and an analysis of any potential contamination (through a comparison of group two and group three).

Depressed participants were randomized to 'collaborative care'--case manager-coordinated medication support and brief psychological treatment, enhanced specialist and GP communication--or a usual care control. The primary outcome was severity of depression (PHQ-9 [20]). In all, 114 participants were recruited, 41 to the intervention group, 38 to the patient randomized control group, and 35 to the cluster-randomized control group. For the intervention compared to the cluster control, the PHQ-9 effect size was 0.63 (95% CI 0.18 to 1.07). There was evidence of substantial contamination between intervention and patient-randomized control participants, with less difference between the intervention group and patient-randomized control group (-2.99, 95% CI -7.56 to 1.58, p = 0.186) than between the intervention and cluster-randomized control group (-4.64, 95% CI -7.93 to -1.35, p = 0.008).

From this 'trial platform' study, we aimed to develop a larger scale phase IV clinical multi-centred trial (which, given the results reported above, would subsequently require to be a cluster randomised design). We recognised early in the conception of the project that a considerable amount of work would be required to optimize an intervention originally designed in the US for a British setting, and we thus aimed to collect extensive qualitative data at different stages of the process.

### Aims of this study

In this paper, we will apply the NPM to our process data in order to consider what we can learn about the additional or 'hidden' work (*i.e*., that which is not immediately apparent at conception of the project or not usually included in publication of results of a trial) that needs to be done to make a collaborative care intervention for depression in primary care both workable and integrated into routine practice in both our forthcoming full-scale trial of collaborative care for depression in the UK and the wider healthcare settings following the trial. In initiating this task, we were particularly interested in the value of application of the NPM to process data in order to aid us in further development and evaluation of this intervention in the UK. This is a novel approach which has not, to our knowledge, yet been widely adopted for use at different stages in the formal analysis of a complex intervention to inform further iterations of the research process, even though it was originally intended for purpose [11].

## Methods

**Figure 2:**
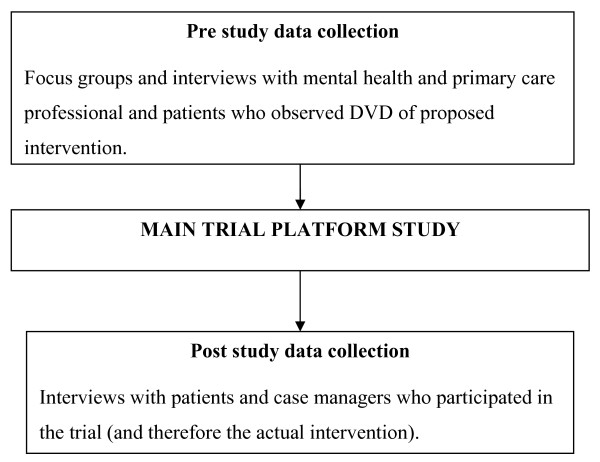
**Study design**.

Application of the NPM to data collected in both focus groups and one-to-one interviews with both practitioners and patients before and after an exploratory randomised controlled trial of a collaborative model of care for depression (see Figure 2).

We utilized the data to both understand and identify the 'hidden work' essential to optimizing the intervention not apparent at the conceptualisation and design stage of the intervention and make predictions about future issues in implementation. Key questions (see table 1) relevant to the specific intervention were derived from the four factors of the NPM by the lead author, who had previous of experience of working with the NPM and discussed with the wider research team. They were considered to be highly relevant to the requirements of the study and no further iterations were required. We considered that data collected before the trial would enable us to test out the predictive value of the NPM in terms of what actually happened in the trial platform, and the data collected after the trial would be of particular value in revising the content of the intervention in terms of both the forthcoming large scale trial and wider dissemination.

### Before the trial

The detailed description of protocol development can be found elsewhere [17]. We identified key prototype collaborative care components using a systematic review and meta-regression [21]. We used qualitative methods to provide a contextualized picture of the views of key stakeholders on the acceptability, feasibility, and barriers to collaborative care for depression in the UK.

### Sample

A convenience sample of stakeholders was recruited from primary care organizations (PCOs) in the north of the UK. Primary Care Physicians (PCPs) and practice nurses were recruited from practices in PCOs that had agreed to participate in the trial. Other participants were recruited from teams and specialist care providers that provided primary and secondary mental healthcare to the PCOs. Patients were recruited by four participating PCPs who each mailed a letter to 20 of their patients who were receiving treatment for depression in primary care. No participants had had any experience of this method of organizing care and none been involved in the trial design.

We interviewed 49 participants. All 38 professionals who were asked to participate in the study agreed to do so: 12 PCPs, four psychiatrists, four clinical psychologists, four practice nurses and 14 mental health workers (seven mental health nurses, two counsellors, three graduate mental health workers, one social worker, and one unqualified support worker). We had planned to conduct focus groups with all respondents, but to arrange mutually convenient times for separate groups of GPs, professionals, and patients proved to be impossible. Therefore, most interviews were conducted individually apart from two focus groups with 11 of the 14 mental health staff. From the 80 letters posted to patients, 17 consented to participate of which 11 were interviewed, five subsequently declined or could not be contacted, and one became so distressed that the interview was abandoned on ethical grounds and the patient was encouraged to contact the PCP.

### Data collection

We had earlier filmed four role-plays, representing those key clinical features of collaborative care that could be represented on film. These included the necessity for brevity of contact in this therapeutic approach (not hour-long sessions); use of telephone consultations; the need for a patient-centered, collaborative approach to care, problem-focused interviewing style; information giving; the skills of medication management and behavioural activation (BA)--an evidence-based psychological treatment for depression [22], which has been used in the low-intensity manner required for short patient contacts in collaborative care. Participants were sent a copy of this videotape/DVD to view prior to their interview.

Interviews and focus groups were structured using an identical topic guide. Although topic areas were similar for patients and professionals, questions to patients focused on their views about potential receipt of the intervention, whereas questions to professionals focused on delivering it. The interviews lasted approximately 30 to 40 minutes, while the focus group activity lasted 1 to 1.5 hours.

### After the trial

We carried out a further round of one-to-one interviews with case managers and patients who have received the intervention..

### Sample

All of the eight case managers from the trial--five graduate mental health workers, one counsellor, two mental health nurses(who had both participated in the pre-trial focus groups)--and 13 of the patients who had been in receipt of the intervention were purposively selected for age, gender, and profession of case manager. We were unable to obtain interviews with the PCPs involved in the study due to our resource pressures.

### Data collection

Data were collected using semi-structured interviews. Patients were asked what they thought about how the intervention was structured, the relationship with the case manager, what they learned about depression from the intervention, and their views about the different elements of the intervention. They were also asked if contacts with, or attitude to, their PCP had altered as a consequence of their involvement in the trial, and whether their symptoms improved or not as a result of their participation. The detailed findings from the patient interviews are reported elsewhere [19].

Case managers were asked how the study protocol differed from their usual approach to assessment and treatment, whether they had any problems with adapting their style of working at all, either in specific ways or with specific clients. We also explored whether there was anything about the protocol that they found beneficial or difficult, and what was its impact on both professional and patient. We asked how easy or difficult it would be for them to adapt their routine way of working to the Collaborative Care for Depression protocol, and the personal and organisational barriers that might exist. Finally, we requested their views on the written materials and the supervision they had received.

### Analysis of the data for this paper

Data analysis was led by the lead author. Two sets of data were entered into the analysis: the pre-trial data that was obtained from interviews and focus groups with participants who observed the DVD of role-played examples of the intervention and discussed what 'might' happen in trying to adapt the intervention; and post-trial data with patients and practitioners who enacted the intervention in the trial platform study.

LG coded the data utilising a simple template [23] or *a priori *coding manual specifically derived from May's original description of the NPM [11], with specific questions derived from the four factors to address implementation of collaborative care for depression (see table 1). This was then entered onto MAXqda2 qualitative analysis software [24]. A total of 61 transcripts (consisting of 59 individual interviews and two focus groups) were included in the analysis. Only data that could be coded according to the NPM-derived template was considered. The findings were discussed in detail with the trial research team, and underwent subsequent revisions to achieve consensus that they accurately reflected the original data and the lessons to be learned from the study for future implementation.

## Results

We will present the findings as they relate to the questions derived from the four factors of the NPM. Several of these arose in relation to the factors in the pre-trial interviews and focus groups that we had not previously considered (and thus were 'hidden work' that needed to be considered and carried out to make the study work that not apparent at the time of trial design), and others arose during the study (and were not predicted in the pre-trial interviews and focus groups) and thus were 'hidden' until participants had direct experience of this kind of intervention, and will inform future work.

### Interactional workability of collaborative care

#### How does collaborative care for depression impact on basic communication, clinical care, and treatment at the level of patient and professional?

The specific communication and confidentiality issues that might and did indeed arise in telephone interviews were successfully predicted beforehand:

'I'd see difficulties with not being able to pick up on cues.' (mental health nurse, before trial)

'Maybe the interviewer would have to say 'I'd like to speak to you now but some of the questions might be quite sensitive. Could you tell me where you are? Are you alone? Are you happy to speak?" (psychiatrist, before trial)

Also predicted was the always potentially difficult task of 'ending' a brief therapeutic relationship:

'It should be an open-ended thing. I don't think treatment should stop, but it would be 'I won't be ringing you up now--but I'll ring you in six month's time, but you can always ring me if there are any issues'. I'd say probably [end] over two to three months--but it would be between the case worker and the patient.'(Patient two, before trial)

Engaging the patient in the process of collaborative care by simultaneously building up trust, but also explaining the systematic and collaborative nature of this approach to care, with regular structured assessments of progress, was challenging, and undoubtedly easier if the first contact was face-to-face:

'They wanted...counselling, even though I did emphasize that I wasn't counselling,...it took time, to build up trust with them.' (case manager graduate worker, after trial)

'I think it was important that they went through the standard set of questions every time they spoke. I felt that everything was explained to the client, why he was doing what he was doing--it was very much 'I'll work together with you' rather than 'I'm just another professional that wants to get rid of you.' (patient eight, after trial)

Postal preparation for the telephone session also proved to be important:

'Sometimes it's useful to use diagrams to explain specific things to people and you can't do that over the phone.' (case manager/graduate worker after trial)

We have previously reported the divergence of views in the before-study interviews about the impact of the telephone on the process of care [17]. This divergence of views was also mirrored in our post-study data:

'Probably it was easier speaking over the phone, because I was busy at work and it was very convenient.' (patient.four, after trial)

'It seemed a lot more impersonal on the phone...I know it's daft, but it seems like they care more when you can see them, the reactions on their faces and things.' (patient nine, after trial)

However, given that the collaborative care protocol also had a positive impact on both quantitative and qualitative outcomes [18,19], and was perceived by the case managers as improving access to treatment for people who might otherwise not engage, it could certainly be concluded that it confers an advantage over existing approaches to clinical care. Indeed sceptical professionals were won over to it:

'I always tended to look at using the telephone...well it's a second way, a substandard way of offering a therapeutic intervention...but I suppose my views have changed from being part of this project.' (case manager/mental health nurse, after trial)

'I suppose, [I] learned to listen to what was not being said rather than what was being said and sort of trying a bit more to pay attention to the silences a little. Trying to pick up more when people sounded uncertain or unsure and tones of voices mostly. Yes, it was just a bit different.'(case manager/mental health nurse, after trial)

Some of the case managers adjusted their own clinical styles of working with patients to the trial protocol. Others found this difficult (the counsellor--see below) whilst some, particularly those trained as graduate mental health workers, did not need to do so to any great degree:

'I was fortunate in that the style with which I normally work is very similar, in fact identical to this.' (case manager/graduate mental health worker, after trial)

But others, as we have indicated, questioned some aspects of the protocol as a valid way of interacting with patients, for example when it came to the need for active engagement:

'It's got to be there from the client, they have to want to help themselves to move forward.' (case manager/counsellor, after trial)

The tasks that we identified as necessary work to optimise interactional workability for future studies are summarised in appendix 1.

### Relational integration

#### How does Collaborative Care impact on the way that health professionals relate to each other?

##### Who needs to be involved? How do we inform them and link with them?

Issues that arose in the trial with respect to the need for clarity of arrangements for liaison between patient, PCP, and case manager, and the roles and responsibilities of the specialist supervisors in relation to the PCP were predicted beforehand:

'Different doctors have different approaches. Some don't like anyone else interfering at all, others are fairly open.' (PCP, before trial)

'I would expect to be the person doing the referral, even if we'd discussed it....We might debate it, but I don't think I would argue with somebody with mental health experience saying, 'I'm hearing things that bother me.' I would do the referral.' (PCP, before trial)

We can anecdotally confirm that these did indeed provide to be important. However we were unable to explore these issues in greater depth after the intervention because we did not carry out post-intervention interviews with the PCPs.

##### Does it seem to be the right thing to be doing? Is it perceived as valid and/or useful?

A wide variety of views were expressed before the study about the elements of the protocol [17], with some degree of scepticism about the evidence base:

'I think I'd just go back to the fact that if you're looking at developing these roles, there needs to be good evidence that people are going to benefit from it..... It's got to be a really strong evidence base that it's a good use of time, money etc.' (psychiatrist, before trial)

It was not a surprise to find that case managers described some hostility to the model amongst their colleagues:

'I think the biggest organisational resistance is, that I hear constantly, is 'but what about the underlying themes, what about the core beliefs that reoccur,' and without working on the underlying themes...then what we are doing is, people would say, sticking a plaster over the cracks...when I have presented this at Psychology Awayday...those were the type of comments I got.' (case manager/mental health nurse, after trial)

From the viewpoint of the case managers who participated in the trial, there was a need to adjust the depression focus of the protocol in research practice to the reality of co-morbidity issues in primary care practice:

'There's things like abuse, self-harm, and stuff like that, underlying things that may come up...a protocol on what happens when you cover the more nasty sort of experiences will be an issue. Anxiety...its a protocol for depression,...we get such a mixed picture....' (case manager/mental health nurse, after trial)

The tasks that we identified as necessary work to optimise relational integration for

future studies are summarised in appendix 2.

### Skill-set workability

#### Does this mean health professionals learning new skills or doing things differently? Is there a person available with the right set of skills to implement Collaborative Care?

Before the trial experienced mental health professionals had strong views about who would be qualified to carry out the role:

Interviewer: 'What kind of people do you see as case managers?'

CPN [community psychiatric nurse]: 'Oh, CPNs. Definitely CPNs! Social workers. Anybody but primary care mental health workers.' (case manager/mental health nurse, before trial)

But some recognised the potential for graduate or new primary care mental health workers [25], who are health or social science (typically psychology) graduates with one further year of training, to fulfil the role of case managers:

'I'm not sure if the graduate workers aren't doing a lot of it already....That's the training that they've been given and that's what they're doing.' (psychiatrist, before trial)

Five of the eight case managers in our study had received such training and two of the other three clearly found it (relatively) easy to adapt their style of working (the counsellor found it more problematic):

'It was the way that we were taught in our training to do that.' (case manager/graduate worker, after trial)

'I felt quite daunted at first, I consider myself to be an experienced mental health professional, but, I felt very strange at first just thinking am I asking all the right questions...and it felt like I was starting again really.' (case manager./mental health nurse, after trial)

#### Does Collaborative Care challenge professional autonomy over working practices? Does it impact on case load and allocation of work?

We observed that working practices at the organisational level sometimes made it difficult for some of the case managers to utilize their skills and/or work to the protocol:

'...our protocol is, you must never ever call a patient from home, ever. If you are going to call a patient you have to call them where you have access to services if something goes wrong. That means that you have to have access to a doctor.' (Case manager/graduate worker, after trial)

It was those case managers who held more senior posts, and not holding the post of graduate mental health worker (*i.e*., not those basically trained in the desired skill set) who had the most freedom to be able to overcome these difficulties because of the relatively autonomous way in which they worked within the organisation:

'I have had to take time, I have been taking time back from phoning people on an evening.' (case manager/mental health nurse, after trial)

In addition, the working practices and governance arrangements concerned with management of risk need to be well developed:

'It needs to cover the area of risk .... They need clear guidance, a protocol to follow and a pathway for each scenario if something happens.' (mental health nurse, before trial)

And the role of supervision predicted in the pre-trial data was confirmed in post-trial interviews with the case managers:

'If you see the case manager as replacing the CPN, which is how I see it, in a way, then the next person up who we need advice from, is going to be the consultant psychiatrist.' (PCP, before trial).

'That was quite nice, being able to have any queries about medication being answered straightaway by a consultant psychiatrist.' (case manager/mental health nurse, after trial)

Given the small number of cases managed by each worker in the trial, the impact on workload was difficult to assess. Key issues for skill-set workability in future studies are summarised in appendix 3.

### Contextual integration

#### Who has the power to make collaborative care for depression happen? Does the system want it to happen? How can we divert resources to it?

Although funding is a major factor in contextual integration, it is not the only issue. The management systems set up to oversee how care is delivered are also crucial and management needs to be capable of facilitating new ways of working. In the pre-trial data, the issue of out-of-hours working to allow for flexibility in contacting people by telephone was not predicted but proved to be an issue. However systems proved inflexible in accommodating this:

'Yes. I'm sure it would be useful to [telephone] in an evening, if it was possible, if that makes sense. If there was a place we could do it. And I suppose there is a reluctance, obviously getting people to work during evenings, just trying to find the workers that are willing to do that late shift if that makes sense.' (Case manager/graduate worker, after trial)

We have noted above the flexibility in working practices that experienced mental health professionals enjoy in the National Health Service (NHS) that therefore makes introduction of new working practices potentially both more and less problematic. Our experienced professionals were free to choose to adopt this model of working, but the same professional freedom for mental health workers makes it harder to impose a new working practice across the board.

Change is especially problematic where professional rivalries surface:

'There are always barriers, because different professionals have views about their own importance and are wanting to protect their 'tribal' interests...It's an issue about who manages who and who has the power--that's always an issue. But not insurmountable.' (psychologist, before trial)

Introduction of any new way of working in an organisation will require effective and informed leadership to manage inter-professional rivalries and the interests of existing services and the development and implementation of a credible business plan:

'There are not a lot of people in primary care at the moment working with depression. So if you take people away to do that, you will be taking them away from doing something else, and I think the resistance will be that the something else is very important, so will it be worth it?' (psychiatrist, before trial)

'Somebody somewhere has to get up in the helicopter and look down and decide what they want. But that's not how it seems to happen. What happens is that people come along and build on sexy new bits of project, to what exists currently. And you end up with a bigger mess than when you started. What you need is for someone to stand back and work out what is really wanted and how it should all be linked together.' (psychologist, before trial)

Issues in optimising contextual integration are addressed in appendix 4.

## Discussion

### The value of the NPM

The NPM provided us with a neat and conceptually rich framework to guide analysis and our thinking about a range of key issues in the implementation of collaborative care for depression in both research trials and routine practice. It provided a novel way of evaluating and interpreting process data that added value to the analysis. Using the model, it was possible to observe that certain predictions about work that would need to be done that could be made from analysis of the pre-trial data relating to the four different factors of the NPM were borne out in the post-trial data. This work was important in our detailed preparation for the trial, although we were still not able to characterise exactly what it involved until completing the trial platform study. Additionally, it may be difficult to predict exactly what work is involved if participants have no experience of a novel intervention, thus we gained some particular insights from post-trial data. In our experience, the importance of doing this work in the preparation for a trial, in order to make a novel intervention work in the setting of a study, is rarely reported with the findings of the trial, and thus this work remains hidden.

### Lessons for collaborative care trials

In our large scale, MRC-funded trial of collaborative care for depression [26], we have learned that it will be essential to address a number of key issues in the preparation of both case managers and supervisors. These include how to engage the patient and explain both the systematic nature of the approach to care (particularly the regular assessment of severity using the PHQ-9) and the time-limited nature of the intervention. There is also a need to address the acquisition of the skills required for telephone working. Clear protocols have been agreed for liaison between professionals and the issue of how to deal with co-morbidities (such as anxiety disorders) has been explicitly addressed.

### Further lessons for wider implementation of collaborative care

The implementation of collaborative care models in the setting of the NHS means that existing relationships, received wisdom about ways of working, and professional roles are challenged, and the organisational tasks required for implementation are considerable but by no means insurmountable. Our findings under the heading of 'contextual integration' will be of particular relevance here. The professional freedom enjoyed by more senior mental health workers in the NHS may work both for and against normalisation of collaborative care as those who wish to adopt new ways of working have the freedom to do so but are not obliged to do so.

### Strengths and weaknesses of the study

Our failure to interview PCPs after the intervention had been delivered was undoubtedly a weakness of the present study. However, we collected a considerable number of interviews in both phases of data collection, and the post-intervention data was not collected from one but four different Primary Care Trusts in the North of England. Nevertheless, we are aware that our participants who were essentially a convenience sample may have been biased, and more open to considering change in practice from more routine and familiar styles of care. We are also aware that we may not have asked all of the important questions of the data, and other researchers may have derived a wider range of questions from application of the four factors of the NPM to this study. This is something that we will revisit in future studies utilizing the model. Additionally, we have not addressed in this research the range of complex issues involved in bringing about organisational change in healthcare, only what needs to be done [27]. Future research might utilise the NPM in addressing the work that is required to implement collaborative care on a much larger scale into a routine healthcare setting, using results of this study in the development of hypotheses that can be tested in the full-scale trial.

### Summary

The NPM provides a useful structure for both guiding and analysing the process by which an intervention is optimized for testing in a larger scale trial or for subsequent wide-scale implementation. Using this framework, we have developed what we hope will be useful guidance for those already implementing collaborative care models, both in the UK, (as part of the Improving Access to Psychological Therapies initiative being led by the Department of Health [28]) and internationally, as it focuses not simply on what are the barriers but what has to be done in practice to make an intervention really work.

## Appendix 1: Optimizing the interactional workability of collaborative care

### 'Work' needs to address

• Engaging the patient.

• Explanation of the systematic nature of approach to care.

• Alliance building- easier if first assessment is face-to-face.

• Explaining the use of the structured approach to assessment of severity.

• Collaborative style of working.

• Specific communication and confidentiality issues raised by telephone working.

• Postal preparation for the telephone session.

• Negotiation of difficult issues raised by ending. Dealing with ambivalence and potential for dependence.

## Appendix 2: Optimizing the relational integration of collaborative care

### 'Work' needs to address

• Clarity of arrangements for liaison between patient, PCP, and case manager.

• Clarification of the roles and responsibilities of the specialist supervisors in relation to the PCP

• Adjusting the depression focus of the protocol in research practice to the reality of co-morbidity issues in primary care practice. Particularly an issue for wider implementation.

• Not only developing the evidence base but educating other key professionals in the wider network about the evidence base for collaborative care.

## Appendix 3: Optimizing the skill-set workability of collaborative care

### 'Work' needs to address

• Recognition within organisations that there is a workforce that is being specifically trained for this task.

• Opportunities for other workers to train in these skills if they wish to.

• Development of comprehensive working protocols to manage risks.

• Appropriate supervision and liaison arrangements.

## Appendix 4: Optimizing the contextual integration of collaborative care

### 'Work' needs to address

• Management practice within the organisation- to facilitate new ways of working.

• Effective service planning.

• Leadership within the local health economy.

• Developing the business case by policy leaders and managers.

## Competing interests

The authors declare that they have no competing interests.

## Authors' contributions

LG conceived and lead the analysis and drafted the paper. DA, JA, AL, and AS carried out the interviews and focus groups. All of the authors contributed to the discussion and interpretation of the findings from the analysis and commented on the paper. DR was the Chief Investigator and grant-holder for the trial. All authors have read and approved the final manuscript.
